# Targeted metabolomics detects a putatively diagnostic signature in plasma and dried blood spots from head and neck paraganglioma patients

**DOI:** 10.1038/s41389-023-00456-4

**Published:** 2023-02-25

**Authors:** Simone De Fabritiis, Silvia Valentinuzzi, Gianluca Piras, Ilaria Cicalini, Damiana Pieragostino, Sara Pagotto, Silvia Perconti, Mirco Zucchelli, Alberto Schena, Elisa Taschin, Gloria Simona Berteşteanu, Diana Liberata Esposito, Antonio Stigliano, Vincenzo De Laurenzi, Francesca Schiavi, Mario Sanna, Piero Del Boccio, Fabio Verginelli, Renato Mariani-Costantini

**Affiliations:** 1 Department of Medicine and Aging Sciences, “G. d’Annunzio” University of Chieti-Pescara, 66100 Chieti, Italy; 2 Center for Advanced Studies and Technology (CAST), 66100 Chieti, Italy; 3 Department of Pharmacy, “G. d’Annunzio” University of Chieti-Pescara, 66100 Chieti, Italy; 4 Otology and Skull Base Unit, Gruppo Otologico, 29121 Piacenza, Italy; 5 Department of Innovative Technologies in Medicine & Dentistry, “G. d’Annunzio” University of Chieti-Pescara, 66100 Chieti, Italy; 6 Department of Oncology-Hematology, Service of Anatomic Pathology, Guglielmo da Saliceto Hospital, 29100 Piacenza, Italy; 7 Familial Cancer Clinic and Oncoendocrinology, Veneto Institute of Oncology, IOV-IRCCS, 35128 Padua, Italy; 8 Carol Davila University of Medicine and Pharmacy, 050474 Bucharest, Romania; 9 Endocrinology, Department of Clinical and Molecular Medicine, Sant’Andrea University Hospital, Sapienza University of Rome, 00189 Rome, Italy

**Keywords:** Cancer metabolism, Tumour biomarkers

## Abstract

Head and neck paragangliomas (HNPGLs), rare chemoresistant tumors curable only with surgery, are strongly influenced by genetic predisposition, hence patients and relatives require lifetime follow-up with MRI and/or PET-CT because of de novo disease risk. This entails exposure to electromagnetic/ionizing radiation, costs, and organizational challenges, because patients and relatives are scattered far from reference centers. Simplified first-line screening strategies are needed. We employed flow injection analysis tandem mass spectrometry, as used in newborn metabolic screening, to compare the plasma metabolic profile of HNPGL patients (59 samples, 56 cases) and healthy controls (24 samples, 24 cases). Principal Component Analysis (PCA) and Partial Least Discriminant Analysis (PLS-DA) highlighted a distinctive HNPGL signature, likely reflecting the anaplerotic conversion of the TCA cycle to glutaminolysis and catabolism of branched amino acids, DNA damage and deoxyadenosine (dAdo) accumulation, impairment of fatty acid oxidation, switch towards the Warburg effect and proinflammatory lysophosphatidylcholines (LPCs) signaling. Statistical analysis of the metabolites that most impacted on PLS-DA was extended to 10 acoustic neuroma and 2 cholesteatoma patients, confirming significant differences relative to the HNPGL plasma metabolomic profile. The best confusion matrix from the ROC curve built on 2 metabolites, dAdo and C26:0-LPC, provided specificity of 94.29% and sensitivity of 89.29%, with positive and negative predictive values of 96.2% and 84.6%, respectively. Analysis of dAdo and C26:0-LPC levels in dried venous and capillary blood confirmed that dAdo, likely deriving from 2′-deoxy-ATP accumulated in HNPGL cells following endogenous genotoxic damage, efficiently discriminated HNPGL patients from healthy controls and acoustic neuroma/cholesteatoma patients on easily manageable dried blood spots.

## Introduction

Paragangliomas (PGLs) are rare, highly vascularized neural crest neoplasms originating from the paraganglia, organelles with neuroendocrine and/or chemoreceptor function(s) associated with the autonomic nervous system across the head/neck and trunk. Based on anatomic location, clinicopathological presentation and mainly parasympathetic (non-chromaffin) versus mainly sympathetic (chromaffin) lineage, PGLs are divided in at least two clinically distinct subsets, *i.e*., head and neck PGLs (HNPGLs), mostly arising from the non-chromaffin paraganglia along the lower cranial nerves or, much less frequently, the sympathetic paravertebral ganglia of the neck, and thoraco-abdominal PGLs (TAPGLs), originating from the chromaffin paraganglia of the thoraco-abdominal region, including the adrenal medulla. Additionally, but rarely, nonchromaffin PGLs arise from the pelvic parasympathetic paraganglia [1, 2]. Germline mutations in one of at least 18 nuclear genes, especially those encoding the 4 subunits of the SDH mitochondrial (mt) enzyme (complex II), i.e., SDHA/B/C/D and its assembly factor SDHAF2 (*SDHx* genes), are associated with at least 35-40% of all PGLs. These mutations may predict higher risk of aggressive disease, of synchronous/metachronous HNPGLs/TAPGLs and of other syndromic tumors that may relate to hereditary PGL [3–6]. Knowledge about PGL genetics impacts on surveillance and follow-up programs for patients and for first/second-degree relatives [6, 7].

Here, we focus on the plasma metabolomics of patients affected with HNPGLs, tumors that preferentially originate from the carotid body or from the parasympathetic paraganglia of the tympanic region and along the jugular vein and the vagal nerve. HNPGLs aggressively infiltrate the adjacent anatomic structures, with severe complications that impair the quality of life and may even be lethal [2, 8–10]. As PGLs in general, HNPGLs are chemo-resistant and radical surgery, difficult in advanced cases, remains the only effective therapy, however targeted peptide receptor radionuclide therapies can control inoperable or progressive disease [11–15]. Metastases are rare but can never be excluded [16, 17]. Anyhow, lifetime follow-up of all patients is granted by the strong genetic basis of PGL, which implies risk of multiple synchronous/metachronous HNPGLs and/or TAPGLs [1]. First/second-degree relatives that result carriers of predisposing mutations identified in index patients should also be regularly controlled [18]. This mandatory follow-up is challenging, because HNPGLs are mostly non-chromaffin and negative screening for plasma or urinary metanephrines is diagnostically irrelevant [8]. Only about one-third of the HNPGLs produce dopamine, determinable by plasma methoxytyramine level and not associated with signs of catecholamine excess [19, 20]. Thus, subjects at high HNPGL risk must be screened with diagnostic scans that expose to ionizing radiation (high-resolution PET/TC), or strong magnetic fields (MRI) [21].

Recently, plasma metabolomics by mass spectrometry (MS) proved useful for the quantification of predetermined tumor markers and for the identification of cancer-associated metabolomic profiles [22–24]. In patients affected with chromaffin PGLs a targeted metabolomic approach demonstrated changes in the concentration of specific amino acids (ornithine, sarcosine, tyrosine, creatinine, histidine, threonine) and lysophosphatidylcholine, that correlated with the results of the urine catecholamines and plasma free metanephrines tests [25, 26].

The present study aimed at characterizing the plasma metabolic profile of HNPGL patients relative to that of healthy controls (HCs) and of patients affected with acoustic neuroma (AN, also known as vestibular schwannoma) or cholesteatoma (CH), common expansive lesions unrelated to HNPGL that originate within the temporal bone. Our approach was based on rapid targeted flow injection analysis (FIA) coupled with tandem MS (MS/MS); a method widely used in newborn screening for metabolic disorders. FIA-MS/MS allows to assess the concentrations of clinically important compounds, including amino acids, organic acids, and fatty acids, in plasma or dried blood spotted on filter paper, the latter adaptable to sampling at home or in general practitioner’s offices and easily deliverable to centralized screening labs by regular mail [27]. Our results outline a distinctive HNPGL metabolomic signature detectable in both plasma and dried blood spots, potentially useful for HNPGL diagnosis and surveillance.

## Results

### Targeted metabolomics distinguish HNPGL patients from healthy controls

**Fig. 1:**
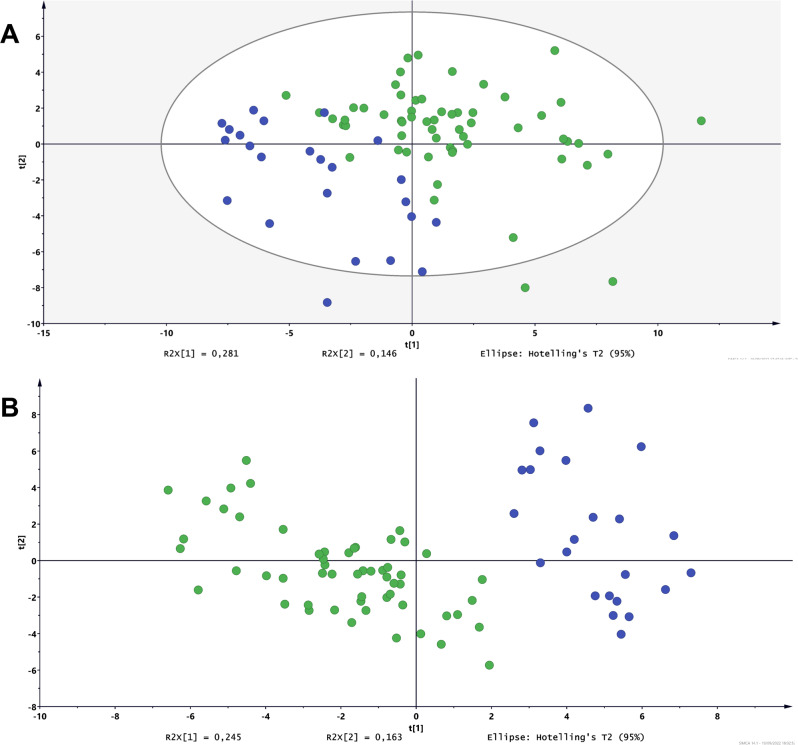
Multivariate analysis of the head and neck paraganglioma patients (HNPGLs) and healthy controls (HCs) based on the metabolites detected by FIA-MS/MS. Panel **A** shows the principal component analysis (PCA) plot with the spatial distribution of the HNPGL patients and the HCs based on similarity. Panel **B** shows the partial least square discriminant analysis (PLS-DA) plot. Only few outliers situate outside the tolerance ellipses. Blue dots: HCs; green dots: HNPGLs.

FIA-MS/MS analysis on plasma quantified 14 amino acids, succinylacetone, 2 nucleosides, free carnitine, 35 acylcarnitines and 4 lysophosphatidylcholines (Table S1). These metabolites were used to conduct multivariate analysis on the HNPGL patients and HCs. Principal components analysis (PCA) and partial least square discriminant analysis (PLS-DA) showed a fair separation between the HNPGL and HC groups. The PCA plot (Fig. 1A) defined the tolerance ellipse based on the Hotelling’s T-squared distribution, which identified 4 outliers (HC24, PTJ146, PTJ150, PV158, Table S2), excluded from subsequent analyses. No significant subclusters based on *SDHx* status, age, sex, Fisch/Shamblin classification, tumor site and embolization status were observed within the HNPGL cluster (Tables S3-S4).

The PLS-DA plot (Fig. 1B) confirmed the spatial distribution outlined by PCA and, considering the first two components, yielded a value of 0.82 for R2Y, which measures goodness of model-to-data fit, and 0.77 for Q2, which assesses consistency between original and predicted data. These values were close to 1, providing robust proof that the PLS-DA model fits the training set and predicts class membership (Y variable). All the R2 and Q2 values to the left of the permutation test plot were lower than the original points to the right of the Q2 regression line, whose vertical intercept was below zero (R2 = 0.0, 0.184; Q2 = 0.0, -0.239), supporting model reliability. The closeness of the R2 and Q2 values obtained by permutation test also suggests that the model explains and predicts the variation of X (metabolite levels) and Y (HNPGL versus HC classification). Specifically, the intercept values were 0.0, 0.184 for R2 and 0.0, -0.239 for Q2 (Fig. S1).

### Metabolites affecting the separation of HNPGL patients from healthy controls

The variable importance in the projection (VIP) plot, which lists in descending order the most significant metabolites, highlights the loadings relevant for the modeling of Y, *i.e*., sample class (HNPGL versus HC), identified by VIP values ≥ 1 (Fig. S2). Specifically, excluding the loadings with VIP < 1, the metabolites taken into account were: Glu, C10:2, C26:0-LPC, d-Ado, C16:1, C24:0-LPC, C18:1, Gln/Lys, C18:2OH, C18:2, C18, C10:1, C5DC/C6OH, C16, C10, SA, Arg, C14, C2, C16:1OH/C17, Val, Leu/Ile/Pro-OH, C18:1OH, Orn, C26 and C8.

**Fig. 2:**
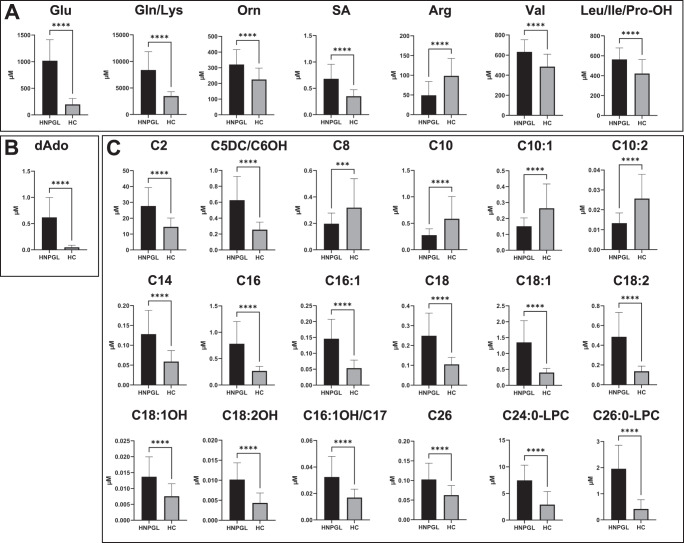
Bar charts for the metabolites with VIP values > 1 in the head and neck paraganglioma patients (HNPGLs) versus the healthy controls (HCs). Data are presented for amino acids (**A**), adenosines (**B**), acyl-carnitines and lysophosphatidylcholines (**C**). Significance was established by t-test (***p < 0.001; ****p < 0.0001).

Glutamate (Glu) was the amino acid with highest VIP, indicating a major impact on clustering. This was confirmed by statistical analysis, as Glu plasma levels were significantly higher in the HNPGL patients than in the HCs (*p* < 0.0001, Fig. 2A). Correspondingly, the plasma concentrations of glutamine (Gln) plus lysine (Lys), isobar species indistinguishable through FIA-MS/MS, of ornithine (Orn), metabolically related to Gln, and of succinylacetone (SA) were significantly higher in the HNPGL patients (*p* < 0.0001 in all cases, Fig. 2A). The amino acids with VIP value < 1 and plasma levels significantly higher in the HNPGL patients included alanine (Ala) (*p* = 0.0353), glycine (Gly) (*p* = 0.0454), methionine (Met) (*p* = 0.0158), phenylalanine (Phe) (*p* = 0.0006), proline (Pro) (*p* = 0.0306) and tyrosine (Tyr) (*p* = 0.0494). The ratios of Leu/Pro (*p* = 0.004) and Phe/Tyr (*p* = 0.0116) were also significantly higher in the HNPGL group (Fig. S3). Intriguingly, arginine (Arg) presented significantly lower levels in the HNPGL group (*p* < 0.0001), while valine (Val) and the pooled isobars leucine (Leu), isoleucine (Ile) and hydroxyproline (Pro-OH) resulted lower in the HC group (*p* < 0.0001, Fig. 2A). Regarding nucleosides, 2-deoxyadenosine (dAdo) emerged as fourth impacting variable because of its high VIP value, consistent with the significantly higher dAdo levels in the HNPGL patients (*p* < 0.0001, Fig. 2B).

Among the short-chain acyl-carnitines (SCACs), the plasma levels of C2 and of the combined isobars C5DC/C6OH resulted significantly higher in the HNPGL patients (*p* < 0.0001 in all cases, Fig. 2C). Several medium/long-chain acyl-carnitines (MCACs/LCACs), including the MCACs C8, C10, C10:1, C10:2 and the LCACs C14, C16, C16:1, C18, C18:1, C18:2, C18:1OH, C18:2OH, C16:1OH, C17, showed an inverse behavior, with significantly higher plasma levels in the HCs (*p* < 0.0001 in all cases, except C8, *p* = 0.0005, Fig. 2C). Notably, C10:2 was associated with the second highest VIP value in the multivariate analysis. Instead, the very long-chain acyl-carnitine (VLCAC) C26 and the lysophosphatidylcholines (LPCs) C24:0-LPC and C26:0-LPC, the latter clearly originating from C26, exhibited higher concentrations in the plasma samples of the HNPGL patients (*p* < 0.0001 in all cases, Fig. 2C).

Bar charts of the metabolites whose plasma concentrations significantly differed in the HNPGL patients versus the HCs (VIP values < 1) are displayed in Fig. S3. Among the acyl-carnitines, C22 presented lower levels in the HNPGL group (*p* = 0.0098), while C5 (*p* = 0.0058), the pooled isobars C3DC and C4OH (*p* < 0.0001), C6 (*p* < 0.0001), C6DC (*p* < 0.0001), C14:2 (*p* = 0.0337), C18OH (*p* = 0.0029) and C24 (*p* = 0.0186) resulted higher in the HNPGL group. Among the LPCs, only C22:0-LPC showed higher levels in the HNPGLs (*p* = 0.0003).

### HNPGL versus acoustic neuroma and cholesteatoma

**Fig. 3:**
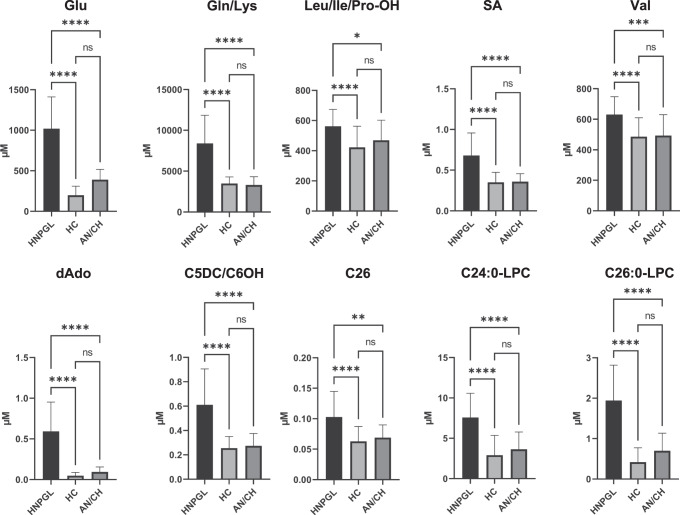
Bar charts for the metabolites that most impacted on the partial least square discriminant analysis (PLS-DA) model. Bar charts show the plasma concentrations of the metabolites with VIP values > 1 in head and neck paraganglioma patients (HNPGLs), healthy controls (HCs), and patients affected with acoustic neuroma or cholesteatoma (AC/CH). Only the metabolites that presented significant differences by t-test are shown (*p < 0.05; **p < 0.01; ***p < 0.001; ****p < 0.0001).

Statistical analysis of the plasma concentrations of the metabolites that most impacted on the PLS-DA model (VIP values > 1) was expanded to include 10 AN and 2 CH patients (Table S3). Significant differences were confirmed for Glu (*p* < 0.0001), Gln/Lys (*p* < 0.0001), Leu/Ile/Pro-OH (*p* = 0.0169), SA (*p* < 0.0001), Val (*p* = 0.0002), dAdo (*p* < 0.0001), C5DC/C6OH (*p* < 0.0001), C26 (*p* = 0.0017), C24:0-LPC (*p* < 0.0001) and C26:0-LPC (*p* < 0.0001) (Fig. 3), suggesting that these metabolites may define an HNPGL-specific metabolic signature. The other metabolites with high VIP values in the original PLS-DA model did not show statistically significant differences in the comparison with the AN/CH patients, suggesting that they participate in a metabolic signature associated with a range of proliferative/inflammatory skull base lesions.

### Plasma metabolite levels versus HNPGL site, stage and SDHx status

We investigated whether the plasma levels of the above-mentioned HNPGL-associated metabolites could be influenced by tumor site, stage and *SDHx* status. We first compared the plasma levels of the relevant metabolites (i.e.: Gln/Lys, Glu, Leu/Ile/Pro-OH, SA, Val, dAdo, C5DC/C6OH, C26, C24:0-LPC, C26:0-LPC) in the various subtypes of HNPGL investigated in this study (tympanic, tympanojugular, vagal and carotid body). No significant differences were observed, except for C5DC/C6OH that showed lower levels in vagal compared to carotid body (*p* = 0.0192) or tympanic HNPGLs (*p* = 0.0271) (not shown). To investigate possible relationships with stage, we considered only tympanic and tympanojugular HNPGL patients, accounting for the largest subset of our case series and uniformly staged accordingly to the Sanna’s modified Fisch classification (Table S4). We compared the concentrations of the HNPGL-associated metabolites in patients with low (A1 to B3) versus high (C3-C4) Sanna’s modified Fisch stage. Statistical analysis did not demonstrate significant differences. Considering mutational status, only Gln/Lys plasma levels differed significantly between *SDHx* carriers and *SDHx* noncarriers (*p* = 0.0041) (Fig. S4).

### Putative HNPGL biomarkers

**Fig. 4:**
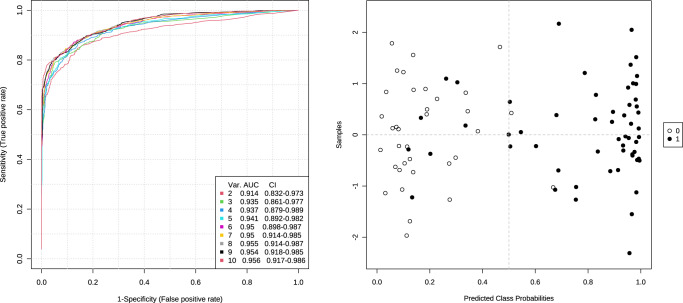
ROC curve-based multivariate analysis for the variables with VIP values > 1 that were statistically significant for the distinction of head and neck paraganglioma (HNPGL) patients from healthy controls (HCs) and acoustic neuroma/cholesteatoma (AN/CH) patients. To the left, cumulative curves based on 2, 3, 4, 5, 6, 7, 8, 9 and 10 metabolites respectively, with area under the curve (AUC) and confidence interval (CI) values. To the right, confusion matrix after cross-validation of the cumulative ROC curve obtained with dAdo and C26:0-LPC. The HNPGL and non-HNPGLs samples, inclusive of HCs and AN/CH, are displayed as black and white dots, respectively.

A ROC curve-based multivariate analysis was conducted for the variables with VIP values > 1 that significantly discriminated among the three test groups (HNPGL patients, HCs, AN/CH patients). The analysis considered HNPGL versus non-HNPGL cases (inclusive of HCs and AN/CH patients). ROC analyses obtained with different numbers of metabolites (2, 3, 4, 5, 6, 7, 8, 9,10) always yielded a large area under the curve (AUC), indicating ability to distinguish between diagnostic groups (Fig. 4). The best confusion matrix obtained from the ROC curve based on 2 metabolites, dAdo and C26:0-LPC, provided a measure of classification accuracy, detecting correct and incorrect predictions for class after cross-validation. An error of 14.3% was observed for prediction of HNPGL class (labeled 1), as 8 patients were misclassified, whereas an error of 5.7% occurred for the definition of non-HNPGL class (labeled 0), as 2 AC/CH patients were misclassified (Fig. 4). These misclassifications cannot be explained based on the information available in the current study (age, sex, tumor site, stage, *SDHx* mutational status).

The 2 metabolites selected by multivariate ROC curve analysis were manually combined to create biomarker models by logistic regression algorithm. Having established that dAdo and C26:0-LPC were noncollinear based on the values of their variance inflation factor (VIF = 2.8) and coefficient of determination (R2 = 0.64), the logistic regression model provided the following equation:

$${{{\mathrm{logit}}}}\left( {{{\mathrm{P}}}} \right) = \log \left( {{{{\mathrm{P}}}}/\left( {1 - {{{\mathrm{P}}}}} \right)} \right) = - 4.296 + 20.04\;{{{\mathrm{dAdo}}}} + 1.532\;{{{\mathrm{C}}}}26\!:\!0 {\hbox{-}} {{{\mathrm{LPC}}}}$$

where P is Pr(y = 1 | x). The classification cutoff for the predicted P is 0.5. The specificity and sensitivity values of the logistic regression model based on dAdo and C26:0-LPC were 94.3% and 89.3%, with positive and negative predictive values of 96.2% and 84.6%, respectively. The estimated AUC was 0.9704 (0.9399 ~ 1). The performance of the logistic regression model is shown in Table S5. AUC, sensitivity, and specificity values for dAdo and C26:0-LPC used separately and in combination are shown in Table S6.

### dAdo and C26:0-LPC in plasma versus dried venous and capillary blood spotted on paper

**Fig. 5:**
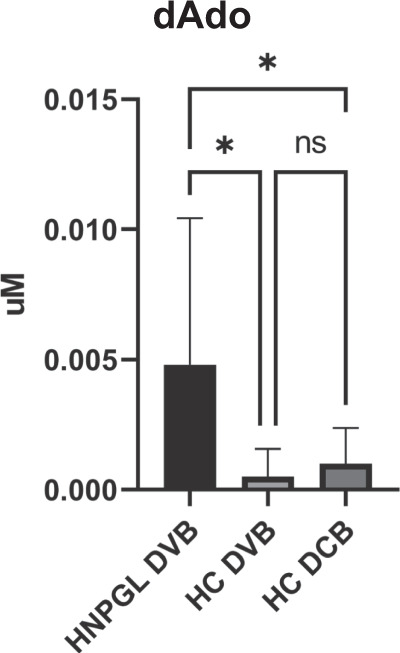
Comparison of dAdo levels in dried venous blood (DVB) versus dried capillary blood (DCB) from head and neck paraganglioma (HNPGL) patients and healthy controls (HCs). Statistically significant differences were observed between the DVBs from the HNPGL patients and the DVBs from the HCs. The levels of dAdo in the DVBs and paired DCBs from the HCs were comparable. Significance was established by ANOVA (HNPGLs: 21 cases; DVBs from HCs: 9 cases; DCBs from HCs: 16 cases) (*p < 0.05).

The analysis of dAdo and C26:0-LPC in plasma versus paired dried venous blood spotted on paper (DVB, *n* = 21 samples) revealed a moderate correlation between dAdo plasma and DVB levels, supported by Pearson r (0.49) and *p* value (0.0259), while no correlation emerged for C26:0-LPC. Finally, dAdo levels were assessed in DVBs from HNPGL patients and HCs versus dried capillary blood (DCB) samples, also spotted on filter paper, from paired HCs. This confirmed that the levels in the DVB and paired DCB samples were comparable, while there were statistically significant differences between the DVBs from the HNPGL patients and the DVBs (*p* = 0.034) and the DCBs (*p* = 0.017) from the HCs (Fig. 5).

## Discussion

To our knowledge, this is the first metabolomic study of HNPGL patients. We exploited FIA-MS/MS, a validated method widely used in newborn screening for inborn errors of metabolism [27]. Using this approach, applicable to both plasma and whole dried blood spotted on filter paper, easy to prepare, preserve and ship, we highlight a putative metabolomic signature of HNPGLs, elegantly supported by clustering in the validated PLS-DA model, that reflects both the biological uniqueness of the samples and their substantial diversity relative to the control group. Based on their high VIP values, 26 different metabolites were identified as variables affecting the model, including dAdo, 7 amino acids, all involved in the urea and reverse TCA cycles, 16 acylcarnitines and 2 LPCs. Quantitative data analysis of these metabolites hints at possible metabolic mechanisms deregulated in HNPGLs.

Low arginine and high ornithine concentrations suggest urea cycle blockage at ornithine transcarbamylase (OTC), consistently with the observed high glutamate levels, likely deriving from transamination reactions involving oxaloacetate from the TCA cycle [28]. OTC downregulation was reported in hepatocellular carcinoma [29], which shares HIF signaling activation with PGL [30]. High glutamate is coherent with the observed increase in branched-chain amino acids (Val and combined Leu, Ile and Pro-OH), whose catabolism contributes to the biosynthesis of nucleotides and non-essential amino acids through the mTOR/glutamate/glutamine axis, and supplies succinyl- and acetyl-CoA to feed the TCA cycle, lipogenesis, and histone acetylation [31–33]. In this respect, HIF2A, the HIFA isomer preferentially activated in HNPGL [34], promotes glutaminolysis [35], a feature of the PGL metabolic phenotype [36, 37].

FIA-MS/MS does not allow direct glutamine quantification, but we hypothesize that the high Gln/Lys values reflect glutamate/glutamine conversion by glutamine synthetase and/or lysine catabolism, consistent with the high levels of glutaryl carnitine (C5DC). Glutamine is the major substrate of the reductive anaplerotic TCA cycle [29, 38] typical of SDH-deficient PGL cells [30], which incorporate glutamine to support anabolism and buffer the leakage of reactive oxygen species (ROS) via glutamate and glutathione (GSH) production [39]. High succinyl-acetone fits into this picture, being a metabolic feature of HIF-dependent cancers, such as HCC [40]. Acyl-carnitines serve as carriers of long-chain fatty acids to the inner mt membrane for β-oxidation, as donors of acyl groups and as source of carnitine involved in the biogenesis of the mt membranes [41]. These functions are strongly deregulated in PGLs, where there is a sharp increase in mt mass accompanied by lipid droplets accumulation [42, 43]. Deregulation of acyl-carnitines has been implicated in various cancers, again including HCC, where it presents a specific pattern, characterized by decrease in SCACs/MCACs, and increase in LCACs [44]. High LCAC levels, as observed in the HNPGL patients, suggest impairment of carnitine palmitoyl-transferase 2 activity, implicated in STAT3-dependent tumorigenesis [44]. High acyl-carnitines were reported in pro-inflammatory conditions, both non-neoplastic, including diabetes, heart failure, and sepsis, and neoplastic, particularly breast cancer, where they were connected to the Warburg effect and to upregulated oxidation of fatty acids and branched amino acids [45]. A pro-inflammatory metabolomic signature is also consistent with the upregulation of phospholipids linked to the expression of proinflammatory cytokines such as C24:0 and C26:0-LPCs [46].

Univariate statistical analysis of the metabolites that most impacted on the PLS-DA model was also carried out on some patients affected with acoustic neuroma and cholesteatoma, common expansive lesions of the skull base that are unrelated to HNPGL. This confirmed the above-described trends for Glu, Gln/Lys, Leu/Ile/Pro-OH, SA, Val, dAdo, C5DC/C6OH, C26, C24:0-LPC and C26:0-LPC, highlighting a potentially specific HNPGL metabolic signature, reasonably associated with: 1) anaplerotic conversion of the TCA cycle to reductive glutamine metabolism and catabolism of branched amino acids; 2) DNA damage and toxic dAdo accumulation; 3) impairment of mt fatty acid oxidation (FAO) with the switch towards the Warburg effect; 4) proinflammatory LPC-mediated signaling. These putatively-specific metabolites were combined in multivariate ROC curves to identify leading biomarkers suitable to HNPGL screening and diagnosis. The best classification tool in the model was based on C26:0-LPC and dAdo, the two metabolites that most impacted on clustering.

C26:0-LPC, implicated in microglial dysfunction and brain diseases, is typically elevated in X-linked adrenoleukodystrophy, a hereditary peroxisomal disorder caused by mutations of the *ABCD1* gene, required for the import of very long chain fatty acids into peroxisomes for β-oxidation [47, 48]. Thus, our data point to a major role of peroxisomal dysfunction in HNPGL.

dAdo is a purine deoxyribonucleoside that typically accumulates in subjects with adenosine deaminase (ADA) deficiency, where high dAdo levels support sustained biosynthesis of cytotoxic dATP. This, particularly in B- and T-cells, inhibits ribonucleotide reductase (RNR), required for DNA synthesis, causing severe combined immunodeficiency accompanied, in highly penetrant cases, by multi-organ disease [49]. The distinctively high dAdo blood/plasma levels found in HNPGL patients, who are putatively not ADA-deficient, could reflect ROS-induced genotoxic damage and S phase replication arrest occurring in proliferating HNPGL cells under constitutive metabolic stress. This could result in the accumulation of 2′-deoxy-ATP unspent in DNA replication, whose extra/intra-cellular catabolism would lead to dAdo accumulation [50]. dAdo inhibits pyrimidine synthesis, but its release within HNPGL tissue could be advantageous in terms of immune response inhibition, vascular vasodilation, and metabolic modulation. Accumulation of dAdo may also block transmethylation reactions through the inactivation of S-adenosylhomocysteine hydrolase (SAHH), thus promoting the buildup of the methyltransferase inhibitor S-adenosyl-L-homocysteine (SAH), possibly implicated in the hypermethylated HNPGL phenotype [19]. Furthermore, high 2′-deoxy-ATP/ATP ratios and dAdo availability could facilitate the synthesis of adenosine 2′-diphosphoribose, an endogenous superagonist of the transient receptor potential (TRP) melastatin 2 (TRPM2) plasma membrane cation channel that, under ROS-induced stress, controls Ca2+ fluxes and hence Ca2+ signaling, mitochondrial respiratory uncoupling and immune function [51–53]. Sustained Ca2+ influx and oxidative stress rapidly reach a cytotoxic and apoptotic threshold in normal cells. Conversely, in HNPGL cells, where alternative metabolism implies reduced dependence on the mitochondrial electron transport chain, ROS exposure activates key transcription factors, most notably HIF2A, whose downstream targets support cell survival and proliferation [1–3, 42, 43, 54, 55].

Importantly, by applying a linear equation to the C26:0-LPC and dAdo concentrations, logistic regression correctly identified the HNPGL patients. Considering the observed positive correlation between levels in plasma and in dried venous or capillary blood levels, dAdo quantification alone discriminated the HNPGL patients from the healthy controls and the acoustic neuroma and cholesteatoma patients.

The FIA-MS/MS method is already used worldwide for newborn screening and is fully validated on both plasma and dried blood spotted on filter paper, as we confirm here for HNPGL patients and controls. Dried venous or capillary blood samples give several advantages in terms of sample size, autonomy in sampling, minimal invasiveness, easy handling, and affordable storage. We suggest that this validated method, meeting the requirements of speed (1 min analysis), simplicity, and costs, could be repositioned for high-throughput cancer screening relying on the existing networks of newborn metabolic screening labs. Such repositioning could be particularly important for the HNPGL patients and their relatives at genetic risk, who require lifelong surveillance, presently based on CT and/or MRI scans [8, 54].

The present study has limitations related to sample size which could affect statistical power, particularly in the assessment of possible relationships between metabolite plasma levels and specific HNPGL features such as site, size, stage, and mutational status. Larger confirmatory studies including TAPGLs, which may co-occur with HNPGLs, and other more common head and neck tumors, especially adenocarcinomas and squamous cell carcinomas, are needed to assess the repositioning of newborn metabolic screening by FIA-MS/MS to the screening and follow of HNPGL and possibly TAPGL patients.

## Materials and methods

### Cases, controls, and sample collection

The study was part of research project IG 2020 ID 24501, supported by the Italian Association for Cancer Research (AIRC) and approved by the Ethical Committee of the Regional Health District “Area Vasta Emilia Nord” (AVEN, http://www.ausl.pc.it/comitato_etico/), protocol #2021/0081925. Fasting (12 h) blood specimens were collected before surgery in lavender-top blood tubes (K2-EDTA) at a quaternary skull base and neurotologic center (*Gruppo Otologico* Clinic, Piacenza, Italy), from 56 patients affected with HNPGL (59 samples, as 3 patients were sampled at two metachronous surgical stages), 10 patients with acoustic neuroma (AN), and 2 with cholesteatoma (CH). Fasting blood samples from 24 healthy age-matched controls (HCs) were donated by consenting in-house staff declaring no specific diseases and regularly controlled by institutional health check (Table S2). Informed consent was obtained from all recruited subjects. All the HNPGL plasma samples, except 4 from tympanic HNPGLs, derived from patients who had been embolized approximately 72 h before blood sampling. Blood samples were maintained at ambient temperature, plasma was prepared within 24 h by centrifugation (10 min at 1000 x g speed at room temperature), aliquoted in sterile 1.5 ml Eppendorf tubes and stored at -80 °C until testing. 21 HNPGL patients were tested on both plasma and whole dried venous blood (DVB) samples. The latter were obtained by spotting 50 μL (~1 drop) of fresh blood onto untouched Whatman 903 (W-903) filter paper discs. Spotted filters were dried for 2–3 h under hood at room temperature and saved in sterile envelopes at room temperature until testing. Twenty-four healthy volunteers were selected as controls. Nine (9) individual DVB specimens were obtained as described above from healthy controls. In parallel, samples of dried capillary blood (DCB) obtained by spotting few drops of capillary blood onto W-903 filter paper were donated from 16 healthy controls. Age and sex distribution of the healthy controls (HCs) and of the HNPGL and acoustic neuroma/cholesteatoma (AN/CH) patients are reported in Table S3; essential clinicopathologic and genetic characteristics of the HNPGL patients, including tumor localization, Shamblin class (used here for both carotid body and vagal PGLs according to Sanna M. et al.) [9, 56, 57], Sanna’s modified Fisch class (for tympanic/tympanojugular PGLs) [10, 58] and *SDHx* germline mutation status, are listed in Table S4.

### Genetic characterization

Point mutations and large deletions/rearrangements in *SDHA*, *SDHB*, *SDHC*, *SDHD*, *SDHAF2* and *TMEM127* were assessed by bidirectional Sanger sequencing of the coding regions and splice sites and multiplex ligation-dependent probe amplification (MLPA) [59]. *SDHx*/*TMEM127* status was assessed in 47 out of the 56 HNPGL cases studied (Tables S2 and S4). A putatively pathogenic germline mutation in one of the 6 susceptibility genes was detected in 19/47 cases (40%), including 3 mutated in *SDHA*, 7 in *SDHB*, 3 in *SDHC*, 5 in *SDHD* and 1 in *SDHAF2*. The remaining 28 cases resulted *SDHx*/*TMEM127* noncarriers. Detected variants were classified into pathogenicity classes according to the guidelines of the American College of Medical Genetics and Genomics (ACMG) [60] and the Cancer Variant Interpretation Group UK [61].

### Metabolite extraction from plasma and targeted FIA-MS/MS analysis

The neonatal screening center of G. d’Annunzio University participates in an accredited national network of laboratories that adopts a uniform screening panel covering 57 clinically-relevant metabolites, including 14 amino acids, 2 nucleosides, free carnitine, 35 acyl-carnitines, 4 lysophosphatidylcholines and succinylacetone. Details of the metabolites evaluated and their reference values, deriving from the NeoBase™ 2 Non-derivatized MSMS kit (PerkinElmer Life and Analytical Sciences, Turku, Finland) are reported in Table S1. Metabolites were extracted from plasma specimens using a modified protocol based on the manufacturer’s workflow designed for newborn screening. Briefly, 10 µL of plasma were subjected to protein precipitation by incubation with 125 µL of internal standards (IS) solution (PerkinElmer) for 30 min at 45 °C, 700 rpm (Eppendorf ThermoMixer® C). Proteins were removed after centrifugation (at max speed in an Eppendorf 5424) and clear supernatants (125 µL) were transferred into new vials. An additional 1 h incubation step was required to derivatize succinylacetone (SA). Finally, 100 µL of centrifuged samples were transferred to 96-well plates for injection of 10 µL into the ion source. Acquisition, 1.2 min long injection-to-injection, was carried out on a FIA platform RenataDX Screening System, including a 3777 C IVD Sample Manager and an ACQUITY™ UPLC™ I-Class IVD Binary Solvent Manager, coupled to a Xevo™ TQD IVD tandem quadrupole mass spectrometer (both from Waters Corporation, Milford, MA, USA). The flow gradient for the mobile phase provided by the kit was set as follows: 0.15 mL/min from 0 to 0.170 min; 0.01 mL/min from 0.170 to 0.980 min; 0.7 mL/min from 0.980 to 1.180 min; 0.15 mL/min from 1.180 min to the end. Data were processed by MassLynx™ (IVD) Software V4.2 with NeoLynx™ Application Manager (Waters Corp). Mass spectrometry (MS) parameters and a complete list of the metabolites and their internal standards (ISs) are provided in Table S3. Metabolite extraction from dried venous or capillary blood spotted on filter paper was performed according to PerkinElmer’s protocol using the NeoBase™ 2 non-derivatized MSMS kit. As already described [62–65], samples were punched out into 3.2 mm disks for the extraction of amino acids, nucleosides, free carnitine, acyl-carnitines, lysophosphatidylcholines and succinylacetone. Finally, 10 µL of supernatant were analyzed on the same FIA-MS/MS platform described above, using the same parameters for injection, MS acquisition and raw data processing. The micromolar (µM) concentrations of all tested metabolites are presented in Table S7.

### Statistical analysis

Multivariate analysis was performed on SIMCA® 14.1 (Sartorius Stedim Data Analytics AB). Principal component analysis (PCA) was used to evaluate the clustering pattern and identify outliers. Partial least square discriminant analysis (PLS-DA) was performed to highlight relationships between predictors (X) and dependent (Y) variables, and to discover the loadings most relevant for the modeling of Y – Variable Importance in the Projection (VIP) by focusing on VIPs > 1. The R2Y and Q2 parameters, which respectively measure goodness of fit of the model to the original data and consistency between original and predicted data by cross-validation were used to assess the reliability of the PLS-DA model. R2Y and Q2 values close to 1 indicate reliability and reproducibility of the obtained model outcomes and power to explain and eventually predict the phenomenon under investigation. The PLS-DA model was validated by 100-fold permutation approach. Univariate statistical differences were evaluated by t-test and ANOVA on GraphPad Prism 9.0 using Tukey’s test for multiple comparisons. The Mann-Whitney test was applied to evaluate differences in continuous or ordinal variables, the Chi-square test was employed for categorical variables. Values of *p* < 0.05 were considered significant. Exploratory analysis based on multivariate Receiver Operating Characteristic (ROC) curve (Explorer) was performed on the MetaboAnalyst 5.0 platform (www.metaboanalyst.ca) to detect relevant features and evaluate the diagnostic performance, using the Support-Vector Machine (SVM) algorithm as classification method and the SVM built-in as feature ranking method. According to the subsampling approach for model training, a prediction of class probabilities was computed by cross-validation using the best classifier, identified by the area under the curve (AUC) value. Based on the selection of specific features, a multiple logistic regression model was generated on GraphPad Prism 9.0, evaluating first the correlation matrix to exclude multicollinearity and whether the null hypothesis was rejected by Log-likelihood ratio (G squared). Correlation analysis was performed on GraphPad Prism 9.0.

## Supplementary information

Supplemental Figures and Tables

Supplementary Table S7

## Data Availability

The values of all tested metabolites (µM concentrations) are presented in the Supplementary Table S7. The corresponding authors are available upon request to address specific questions.
