# Supplementary material for: Targeted metabolomics detects a putatively diagnostic signature in plasma and dried blood spots from head and neck paraganglioma patients
Source: Oncogenesis. 2023 Feb 25;12(1):10. doi: 10.1038/s41389-023-00456-4 (PMC9968333; doi:10.1038/s41389-023-00456-4)
Supplement: Supplementary file 1 — Supplemental Figures and Tables [file 41389_2023_456_MOESM1_ESM.pdf]

Supplementary Information

Targeted metabolomics detects a putatively diagnostic signature in plasma and dried blood spots from head and neck paraganglioma patients

Simone De Fabritiis*[1,2], Silvia Valentinuzzi*[2,3], Gianluca Piras[4], Ilaria Cicalini[2,5], Damiana Pieragostino[2,5], Sara Pagotto[2], Silvia Perconti[2], Mirco Zucchelli[2,5], Alberto Schena[6], Elisa Taschin[7], Gloria Simona Berteșteanu[4,8], Diana Liberata Esposito[2,5], Antonio Stigliano[9], Vincenzo De Laurenzi[2,5], Francesca Schiavi[7], Mario Sanna[4], Piero Del Boccio[2,3], Fabio Verginelli[§2,3], Renato Mariani-Costantini [§2]

[1] Department of Medicine and Aging Sciences, "G. d'Annunzio" University of Chieti-Pescara, 66100 Chieti, Italy.

[2] Center for Advanced Studies and Technology (CAST), 66100 Chieti, Italy.

[3] Department of Pharmacy, "G. d'Annunzio" University of Chieti-Pescara, 66100 Chieti, Italy.

[4] Otology and Skull Base Unit, Gruppo Otologico, 29121 Piacenza, Italy.

[5] Department of Innovative Technologies in Medicine & Dentistry, "G. d'Annunzio" University of Chieti-Pescara, 66100 Chieti, Italy.

[6] Department of Oncology-Hematology, Service of Anatomic Pathology, Guglielmo da Saliceto Hospital, 29100 Piacenza, Italy.

[7] Familial Cancer Clinic and Oncoendocrinology, Veneto Institute of Oncology, IOV-IRCCS, 35128 Padua, Italy.

[8] *Carol Davila* University of Medicine and Pharmacy, 050474 Bucharest, Romania.

[9] Endocrinology, Department of Clinical and Molecular Medicine, *Sant'Andrea* University Hospital, *Sapienza* University of Rome, 00189 Rome, Italy.

*These authors contributed equally to this work.

§ Corresponding authors.

***Supplementary Table S1*. Mass spectrometry (MS) parameters used for targeted flow injection analysis (FIA) coupled with tandem MS (MS/MS).** Multiple reaction monitoring (MRM) transition, cone voltage and collision energy (CE) are shown for each analyte. Internal standards (IS) are in brackets. *3-(5-methyl-1H-pyrazol-3-yl) propanoic acid (MPP) is used as IS for succinylacetone (SA)**.**

Abbreviation (ISs)	Analyte	MRM transition	Cone (V)	CE (eV)
**Arg**<br>**($^{2}H_4$, ^{13}C-Arg)**	Arginine	175.1>70.1<br>180.1>75.1	34	21
**Gly**<br>**($^{15}N_2$-^{13}C-Gly)**	Glycine	76.0>30.0<br>78.0>32.0	22	7
**Ala**<br>**($^{2}H_3$-Ala)**	Alanine	90.1>44.0<br>93.1>47.1	22	8
**Pro**<br>**($^{13}C_5$-Pro)**	Proline	116.1>70.1<br>121.1>74.1	28	12
**Orn**<br>**($^{2}H_6$-Orn)**	Ornithine	133.1>70.1<br>139.1>76.1	22	16
**Gln/Lys**<br>**($^{13}C_5$-Gln)**	Glutamine/Lysine	147.1>84.0<br>152.1>88.1	22	16
**Glu**<br>**($^{13}C_5$-Gln)**	Glutamate	148.1>84.0<br>152.1>88.1	24	14
**SA**<br>**($^{13}C_5$-MPP*)**	Succinylacetone	155.1>109.1<br>160.1>114.1	24	22
**ASA**<br>**($^{2}H_4$, ^{13}C-Arg)**	Argininosuccinic acid	291.1>70.1<br>180.1>75.1	42	30
**Ado**<br>**($^{2}H_5$-Ado)**	Adenosine	268.1>136.1<br>273.1>136.1	32	18
**dAdo**<br>**($^{2}H_5$-dAdo)**	2-Deoxyadenosine	252.1>136.1<br>257.1>136.1	29	14
**Cit**<br>**($^{2}H_2$-Cit)**	Citrulline	176.1>113.1<br>178.1>115.1	24	16
**Leu/Ile/Pro-OH**<br>**($^{2}H_3$-Leu)**	Leucine/Isoleucine/Hydroxyproline	132.1>86.1<br>135.1>89.1	24	10
**Met**<br>**($^{2}H_3$-Met)**	Methionine	150.1>104.1<br>153.1>107.1	24	10
**Phe**<br>**($^{13}C_6$-Phe)**	Phenylalanine	166.1>120.1<br>172.1>126.1	25	12
**Tyr**<br>**($^{13}C_6$-Tyr)**	Tyrosine	182.1>136.1<br>188.1>142.1	26	12

Abbreviation (ISs)	Analyte	MRM transition	Cone (V)	CE (eV)
**Val**<br>**($^{15}N_2$-$^{13}C_5$-Val)**	Valine	118.1>72.1<br>124.1>77.1	23	10
**C0**<br>**($^{2}H_9$-C0)**	Free carnitine	162.1>1030<br>171.2>103.0	38	16
**C2**<br>**($^{2}H_3$-C0)**	Acetylcarnitine	204.1>85.0<br>207.1>85.0	34	18
**C3**<br>**($^{2}H_3$-C3)**	Propionylcarnitine	218.1>85.0<br>221.2>85.0	32	18
**C4**<br>**C3DC/C4OH**<br>**($^{2}H_3$-C4)**	Butyrylcarnitine<br>Malonylcarnitine/3-hydroxybutyrylcarnitine	232.2>85.0<br>248.1>85.0<br>235.2>85.0	36	18
**C5**<br>**C5:1**<br>**C4DC/C5OH**<br>**($^{2}H_9$-C5)**	Valerylcarnitine<br>Tiglylcarnitine<br>Methylmalonylcarnitine/3-hydroxyisovalerylcarnitine	246.2>85.0<br>244.2>85.0<br>262.1>85.0<br>255.2>85.0	38	20
**C6**<br>**C6DC**<br>**($^{2}H_3$-C6)**	Hexanoylcarnitine<br>Methylglutarylcarnitine	260.2>85.0<br>290.2>85.0<br>263.2>85.0	37	20
**C5DC/C6OH**<br>**($^{2}H_6$-C5DC)**	Glutarylcarnitine/3-Hydroxy-hexanoylcarnitine	276.2>85.0<br>282.2>85.0	40	24
**C8**<br>**C8:1**<br>**($^{2}H_3$-C8)**	Octanoylcarnitine<br>Octenoylcarnitine	288.2>85.0<br>286.2>85.0<br>291.2>85.0	42	22
**C10**<br>**C10:1**<br>**C10:2**<br>**($^{2}H_3$-C10)**	Decanoylcarnitine<br>Decenoylcarnitine<br>Decadienoylcarnitine	316.2>85.0<br>312.2>85.0<br>314.2>85.0<br>319.3>85.0	45	22
**C12**<br>**C12:1**<br>**($^{2}H_3$-C12)**	Dodecanoylcarnitine<br>Dodecenoylcarnitine	344.3>85.0<br>342.3>85.0<br>347.3>85.0	46	24
**C14**<br>**C14:1**<br>**C14:2**<br>**C14:OH**<br>**($^{2}H_3$-C14)**	Tetradecanoylcarnitine (myristoylcarnitine)<br>Tetradecenoylcarnitine<br>Tetradecadienoylcarnitine<br>Hydroxytetradecenoylcarnitine	372.3>85.0<br>370.3>85.0<br>368.3>85.0<br>388.3>85.0<br>375.3>85.0	52	25

Abbreviation (ISs)	Analyte	MRM transition	Cone (V)	CE (eV)
**C16**	Hexadecanoylcarnitine (palmitoylcarnitine)	400.3>85.0	55	26
**C16:1**	Hexadecenoylcarnitine	398.3>85.0		
**C16:1OH/C17**	Hydroxyhexadecenoylcarnitine	414.3>85.0		
**C16:OH**	Hydroxyhexadecanoylcarnitine	416.3>85.0		
**($^2H_3$-C16)**		403.4>85.0		
**C18**	Octadecanoylcarnitine	428.4>85.0	56	28
**C18:1**	Octadecenoylcarnitine	426.4>85.0		
**C18:2**	Octadecadienoylcarnitine	424.3>85.0		
**C18:OH**	Hydroxyoctadecanoylcarnitine	444.4>85.0		
**C18:1OH**	Hydroxyoctadecenoylcarnitine	442.4>85.0		
**C18:2OH**	Hydroxyoctadecadienoylcarnitine	440.3>85.0		
**($^2H_3$-C18)**		431.4>85.2		
**C20**	Arachidic-carnitine	456.4>85.0	69	34
**C22**	Behenic-carnitine	484.4>85.0		
**C24**	Tetracosanoic-carnitine	512.5>85.0		
**C26**	Hexacosanoic-carnitine	540.5>85.0		
**($^2H_3$-C26)**		543.5>85.0		
**C20:0-LPC**	1-arachidoyl-2-hydroxy-sn-glycero-3-phosphocholine	552.4>104.1	74	30
**C22:0-LPC**	1-behenoyl-2-hydroxy-sn-glycero-3-phosphocholine	580.4>104.1		
**C24:0-LPC**	1-lignoceroyl-2-hydroxy-sn-glycero-3-phosphocholine	608.5>104.1		
**C26:0-LPC**	1-hexacosanoyl-2-hydroxy-sn-glycero-3-phosphocholine	636.5>104.1		
**($^2H_4$-C26:0-LPC)**		640.5>104.1		

Supplementary Table 2. List of the 68 head and neck patients (56 affected with paraganglioma, 10 with acoustic neuroma, 2 with cholesteatoma) and of the 24 healthy controls included in the study. Tumor site, gender, age at surgery and stage are reported for all the head and neck tumor cases, *SDHx* mutational status only for paragangliomas. Only gender and age are reported for healthy controls.

Case code	Tumor site	Gender	Age at surgery	Sanna's Fisch stage[1] or Shamblin class[2]	*SDHx* status	*Acmg[3]/ CanVIG[4] classification*	Evidence for pathogenicity[3]
**PTJ79**	Left tympanojugular	M	40	C2 Di2	Noncarrier[5]		
**PTJ86**	Left tympanojugular	M	15	C1	*SDHD c.27delC p.(Val10Phefs*5)*[5]	*4*	PVS1 very strong, PM2 supporting
**PTJ115**	Right tympanojugular	F	39	C3 De2	*Noncarrier*		
**PTJ116**	Left tympanojugular	M	47	C2 Di1	*SDHC c.251T>C p.(Leu84Pro)*	*3*	PM2 supporting, PP3 supporting
**PTJ117**	Left tympanojugular	F	43	C2	*Noncarrier*		
**PTJ118**	Right tympanojugular	F	16	C3 Di2	*SDHB c.287-1G>C p.?*	*5*	PVS1 very strong, PS3 very strong, PP4 strong, PM2 supporting
**PTJ119[6]**	Right tympanojugular	M	41	C2 Di2	*Noncarrier*		
**PC120**	Left carotid body	F	44	Class II	*Noncarrier*		
**PTJ121**	Left tympanojugular	F	12	C2	*Noncarrier*		
**PTJ122**	Right tympanojugular	M	26	C1	*SDHD c.314+1G>C p.?*	*5*	PVS1 very strong, PS3 very strong, PP4 strong, PM2 supporting
**PTJ123**	Left tympanojugular	F	51	C1	*Noncarrier*		
**PTJ125**	Right tympanojugular	M	50	C2	*Noncarrier*		
**PTJ128**	Left tympanojugular	F	41	C3 Di2	*SDHB c.541-3C>G p.?*	*3*	PP3 supporting
**PTJ130[6]**	Right tympanojugular	M	49	C3 Ve1	*Noncarrier*		
**PTJ131**	Right tympanojugular	F	58	C3 De2	*SDHC c.224G>A p.(Gly75Asp)*	*4*	PP4 strong, PM2 supporting, PP3 supporting
**PT132**	Left tympanic	F	45	A2	*Noncarrier*		

Case code	Tumor site	Gender	Age at surgery	Sanna's Fisch stage[1] or Shamblin class[2]	*SDHx* status	*Acmg[3]/ CanVIG[4] classification*	Evidence for pathogenicity[3]
**PTJ133[6]**	Left tympanojugular	F	37	C4 Di2 Ve2	*SDHB c.286+1G>A p.?*	*5*	PVS1 very strong, PP4 strong, PM2 supporting
**PC134**	Left carotid body	F	39	Class III	*Noncarrier*		
**PTJ135**	Right tympanojugular	M	38	C2	*Noncarrier*		
**PTJ136**	Left tympanojugular	M	44	C3 De1	*Noncarrier*		
**PTJ137**	Right tympanojugular	F	51	B1	*Noncarrier*		
**PTJ140**	Right tympanojugular	F	47	C1 Di1	*Noncarrier*		
**PTJ141**	Right tympanojugular	M	29	C2 De1	*SDHB c.303T>G p.(Cys101Trp)*	*3*	PM1 moderate, PM2 supporting, PP3 supporting
**PTJ142**	Right tympanojugular	F	62	C3	*Noncarrier*		
**PTJ143**	Left tympanojugular	M	21	C2	*SDHD c.305A>C p.(His102Pro)*	*4*	PP4 strong, PM5 moderate, PM2 supporting, PP3 supporting
**PTJ/PC145[7]**	Left tympanojugular + right carotid body	F	34	C2/Class I	*SDHD c.52+2T>C p.?*	*5*	PVS1 very strong, PP4 strong, PM2 supporting
**PTJ146**	Left tympanojugular	M	35	C1	*Noncarrier*		
**PTJ147**	Left tympanojugular	F	53	C2 Di1	*Noncarrier*		
**PTJ148**	Right tympanojugular	F	50	C1	*SDHB c.73-8A>G*	*4*	PS3 strong, PP4 moderate, PM2 supporting, PP3 supporting
**PC149**	Right carotid body	F	55	Class II/III	*SDHAF2 c.233G>A p.(Gly78Glu)*	*3*	PM5 moderate, PP3 supporting, PM2 supporting, PP4 supporting
**PTJ150**	Left tympanojugular	M	50	C2	*Noncarrier*		
**PTJ151**	Right tympanojugular	M	24	C2	*SDHA c.1304 T>A p.(Leu435Gln)*	*3*	PM2 supporting, PP3 supporting
**PC152**	Left carotid body	M	38	Class III	*Noncarrier*		
**PTJ154**	Right tympanojugular	F	25	C2 De1	*SDHA c.1151C>G p.(Ser384*)*	*4*	PVS1 very strong, PM2 supporting
**PT155**	Right tympanic	M	47	A1	*Noncarrier*		

Case code	Tumor site	Gender	Age at surgery	Sanna's Fisch stage[1] or Shamblin class[2]	*SDHx* status	*Acmg[3]/ CanVIG[4] classification*	Evidence for pathogenicity[3]
**PTJ156**	Right tympanojugular	F	32	C2	*SDHB c.546dup p.(Leu183Alafs*11)*	*4*	PVS1 very strong, PM2 supporting
**PTJ157**	Right tympanojugular	M	64	C1	*Noncarrier*		
**PV158**	Left vagal	F	63	Class III	*Noncarrier*		
**PTJ159**	Left tympanojugular	F	46	C2	*Noncarrier*		
**PTJ161**	Right tympanojugular	F	63	C2 De2	*Noncarrier*		
**PT168**	Right tympanic	F	43	B3	*Noncarrier*		
**PC169**	Left carotid body	F	56	NA	*SDHB c.778G>C p.(Gly260Arg)*	*4*	PP4 strong, PM2 supporting, PP3 supporting
**PV170**	Left vagal	F	67	Class III	*SDHA c.1766G>A p.(Arg589Gln)*	*4*	PP4 strong, PM5 moderate, PM2 supporting, PP3 supporting
**PTJ171**	Right tympanojugular	F	27	C2 De1	*SDHD c.52+2T>C*	*5*	PVS1 very strong, PP4 strong, PM2 supporting
**PTJ173**	Right tympanojugular	M	53	C4 Di1 Ve	*SDHC c. (20+1_21-1)_(77+1_78-1)del*	*4*	PVS1 very strong, PM2 supporting
**PTJ175**	Left tympanojugular	F	58	C2 Di2	*Noncarrier*		
**PTJ176**	Left tympanojugular	M	26	C1	NA		
**PTJ177**	Right tympanojugular	M	26	C1	NA		
**PTJ178**	Tympanojugular	F	58	NA	NA		
**PV179**	Right vagal	F	30	Class III	NA		
**PV180**	Left vagal	M	53	Class III	NA		
**PV181**	Right vagal	F	41	Class III	NA		
**PTJ182**	Right tympanojugular	M	53	C2	NA		
**PTJ187**	Left tympanojugular	F	68	C2	NA		
**PT188**	Left tympanic	M	83	A1	*Noncarrier*		
**PTJ189**	Right tympanojugular	M	49	C2	NA		

Case code	Other skull base tumors	Gender	Age at surgery	Hannover[8] or Sanna[9] classification			
**AN1**	Acoustic neuroma	F	62	T4a			
**AN2**	Acoustic neuroma	F	28	T4a			
**AN3**	Acoustic neuroma	M	60	T2			
**AN4**	Acoustic neuroma	M	44	T3a			
**AN5**	Acoustic neuroma	M	43	T3b			
**AN6**	Acoustic neuroma	F	53	T1			
**AN7**	Acoustic neuroma	F	53	T3a			
**AN8**	Acoustic neuroma	F	58	T2			
**AN9**	Acoustic neuroma	M	75	T2			
**AN10**	Acoustic neuroma	M	66	T3a			
**CH1**	Cholesteatoma	M	25	Supralabyrinthine type			
**CH2**	Cholesteatoma	M	45	Massive type			
**Case code**	**Healthy controls**	**Gender**	**Age**				
**HC1**		F	45				
**HC2**		F	51				
**HC3**		M	27				
**HC4**		M	56				
**HC5**		F	53				
**HC6**		M	52				
**HC7**		F	59				
**HC8**		M	65				
**HC9**		M	36				
**HC10**		F	45				
**HC11**		M	70				
**HC12**		M	51				
**HC13**		F	69				
**HC14**		F	48				

**HC15**		M	37				
**HC16**		F	30				
**HC17**		F	30				
**HC18**		F	44				
**HC19**		F	32				
**HC20**		M	51				
**HC21**		M	33				
**HC22**		F	42				
**HC23**		M	52				
**HC24**		F	27				

[1] Sanna's modified Fisch stage for tympanic and tympanojugular paraganglioma is in letters followed by Arabic number according to Prasad SC, et al. Tympanojugular Paragangliomas: Surgical Management and Clinicopathological Features. In: Mariani-Costantini R, Editor: Paraganglioma: A Multidisciplinary Approach. Brisbane (AU): Codon Publications, 2019; pp 99–123.

[2] Shamblin class is in Roman numerals according to Prasad SC, et al. Carotid Body and Vagal Paragangliomas: Epidemiology, Genetics, Clinicopathological Features, Imaging, and Surgical Management. In: Mariani-Costantini R, Editor: Paraganglioma: A Multidisciplinary Approach. Brisbane (AU): Codon Publications, 2019; pp 81–98.

[3] Richards S, et al., Standards and guidelines for the interpretation of sequence variants: a joint consensus recommendation of the American College of Medical Genetics and Genomics and the Association for Molecular Pathology. Genet Med 2015; 17: 405–424.

[4] Garrett A. et al. Quantifying evidence toward pathogenicity for rare phenotypes: The case of succinate dehydrogenase genes, SDHB and SDHD. Genet Med 2022; 24: 41–50.

[5] *SDHx* status previously reported in Verginelli F. et al. Paragangliomas arise through an autonomous vasculo-angio-neurogenic program inhibited by imatinib. Acta Neuropathol 2018; 135: 779–798.

[6] These patients underwent two-staged tumor removals and were sampled at each stage. Reported age is at first stage.

[7] This patient presented with two synchronous paragangliomas (tympanojugular, C2 according to Sanna's modified Fisch stage, and carotid body, Shamblin class I).

[8] Hannover classification is in letters followed by Arabic numbers according to Samii M, Matthies C. Management of 1000 vestibular schwannomas (acoustic neuromas): surgical management and results with an emphasis on complications and how to avoid them. Neurosurgery 1997; 40: 11–23.

[9] Sanna classification for petrous bone cholesteatoma is described in Danesi G, Cooper T, Panciera DT, Manni V, Côté DWJ. Sanna Classification and Prognosis of Cholesteatoma of the Petrous Part of the Temporal Bone: A Retrospective Series of 81 Patients. Otol Neurotol 2016; 37: 787–792.

Supplementary Table S3. Age and sex distribution of the head and neck paraganglioma (HNPGL) and acoustic neuroma/cholesteatoma (AN/CH) patients and of the healthy control (HC) subjects.

	**HNPGL (n=56)**	**HC (n=24)**	**AN/CH (n=12)**	***p*-value HNPGL vs HC**	***p*-value HNPGL vs AN/CH**
**Age (years)**				0.5090	0.1164
**Mean**	43.84	45.88	51.00		
**Median**	44.50	46.50	53.00		
**SD**	14.58	12.75	14.81		
**Sex [n (%)]**				0.8058	0.3287
**Male**	24 (43)	11 (46)	7 (58)		
**Female**	32 (57)	13 (54)	5 (42)		

Supplementary Table S4. Clinicopathological and genetic characteristics of the tested head and neck paraganglioma (HNPGL) patients. Sanna's modified Fisch stage, which reflects anatomic location and tumor size, is given for jugulotympanic and tympanic paragangliomas, Shamblin classification, reflecting carotid vessels involvement, is provided for carotid body and vagal paragangliomas*.

HNPGL characteristics	n (%)
Carotid body	5 (9)
Vagal	5 (9)
Jugulotympanic	41 (73)
Tympanic[1]	4 (7)
Carotid body + Jugulotympanic[2]	1 (2)
Total	***56***
**Sanna's Fisch stage**	**n**
A1	2
A2	1
B1	1
B3	1
C1	9
C2	21
C3	7
C4	2
Total	***44***
**Shamblin class***	**n**
II	1
II/III	1
III	7
Total	***9***
**Sanna's Fisch + Shamblin[2]**	
C2/I	1
**Stage not available[3]**	2
***SDHx* status**	**n (%)**
SDHA	3 (5.4)
SDHB	7 (12.5)
SDHC	3 (5.4)
SDHD	5 (9.0)
SDHAF2	1 (1.8)
Noncarriers	28 (50)
NA[4]	9 (15.9)
Total	***56***

* Shamblin classification is used for both carotid body and vagal paragangliomas following Sanna M, Piazza P, Shin S-H, Flanagan S, Mancini F. Microsurgery of Skull Base Paragangliomas, Chapter 12, pp 455-458, Stuttgart, Thieme, 2013 (doi:10.1055/B-002-66243).

[1] tympanic paragangliomas were the only HNPGLs that did not undergo preoperative embolization.

[2] this category includes a patient with synchronous tympanojugular and carotid body paragangliomas.

[3] this category includes patient(s) with paragangliomas not operated at the Gruppo Otologico clinic in Piacenza and whose classification according to Sanna's modified Fish or Shamblin stage was not available.

[4] not evaluated for germline *SDHx/TMEM 127* mutations.

***Supplementary Table S5*. Performances of the logistic regression model based on dAdo and C26:0-LPC.**

	Estimate	Std. Error	95% CI	Odds ratio
**Intercept**	-4.296	1.105	-6,945 to -2,478	0,01362
**dAdo**	20.04	6.485	9,493 to 35,38	5,04E+08
**C26:0-LPC**	1.532	0.8729	-0,1166 to 3,420	4,628

Supplementary Table S6. Area under the curve (AUC), sensitivity, and specificity values for dAdo and C26:0-LPC used separately and in combination. Values were calculated using the prediction model based on 56 HNPGL and 35 non-HNPGL plasma samples*.

Predictor(s)	AUC	Sensitivity (%)	Specificity (%)
**dAdo**	0.937	82.7	93.3
**C26:0-LPC**	0.880	74.8	96.5
**dAdo + C26:0-LPC**	0.970	89.3	94.3

* Four outliers (3 HNPGL and 1 healthy control samples) were excluded based on the Hotelling's T-squared distribution (see Results).

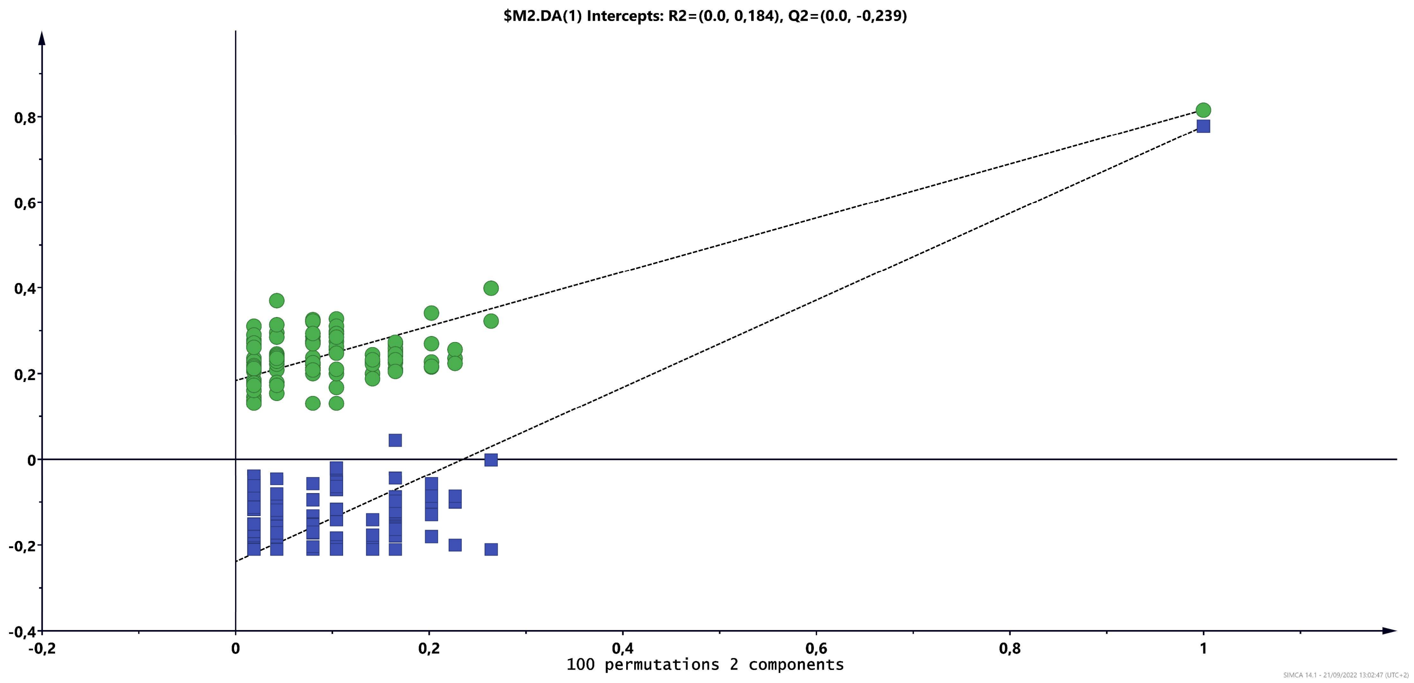

Supplementary Figure S1. Cross-validation plot of the PLS-DA model obtained by 100 permutations, with R2 values in green and Q2 values in blue.

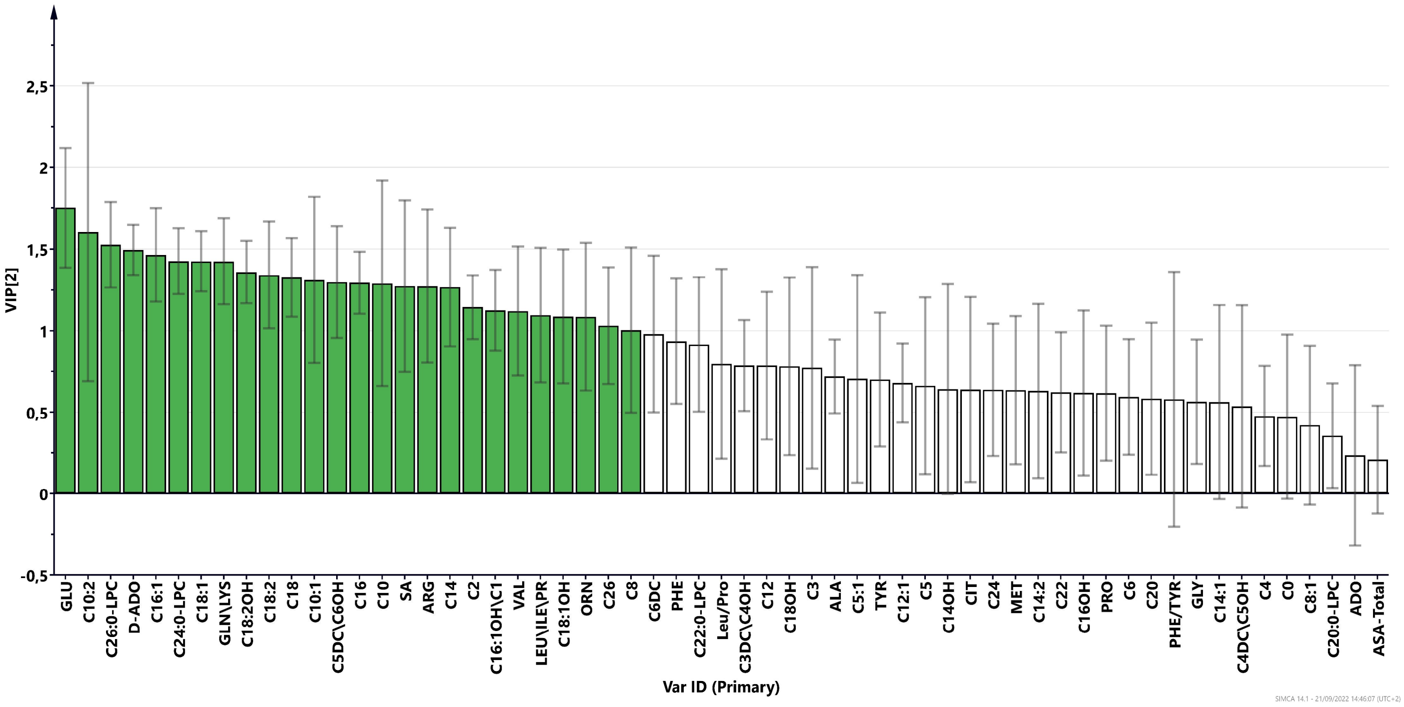

Supplementary Figure S2. Variable importance in the projection (VIP) plot showing the association of the loadings with VIP values as a measure of their impact on the modeling.

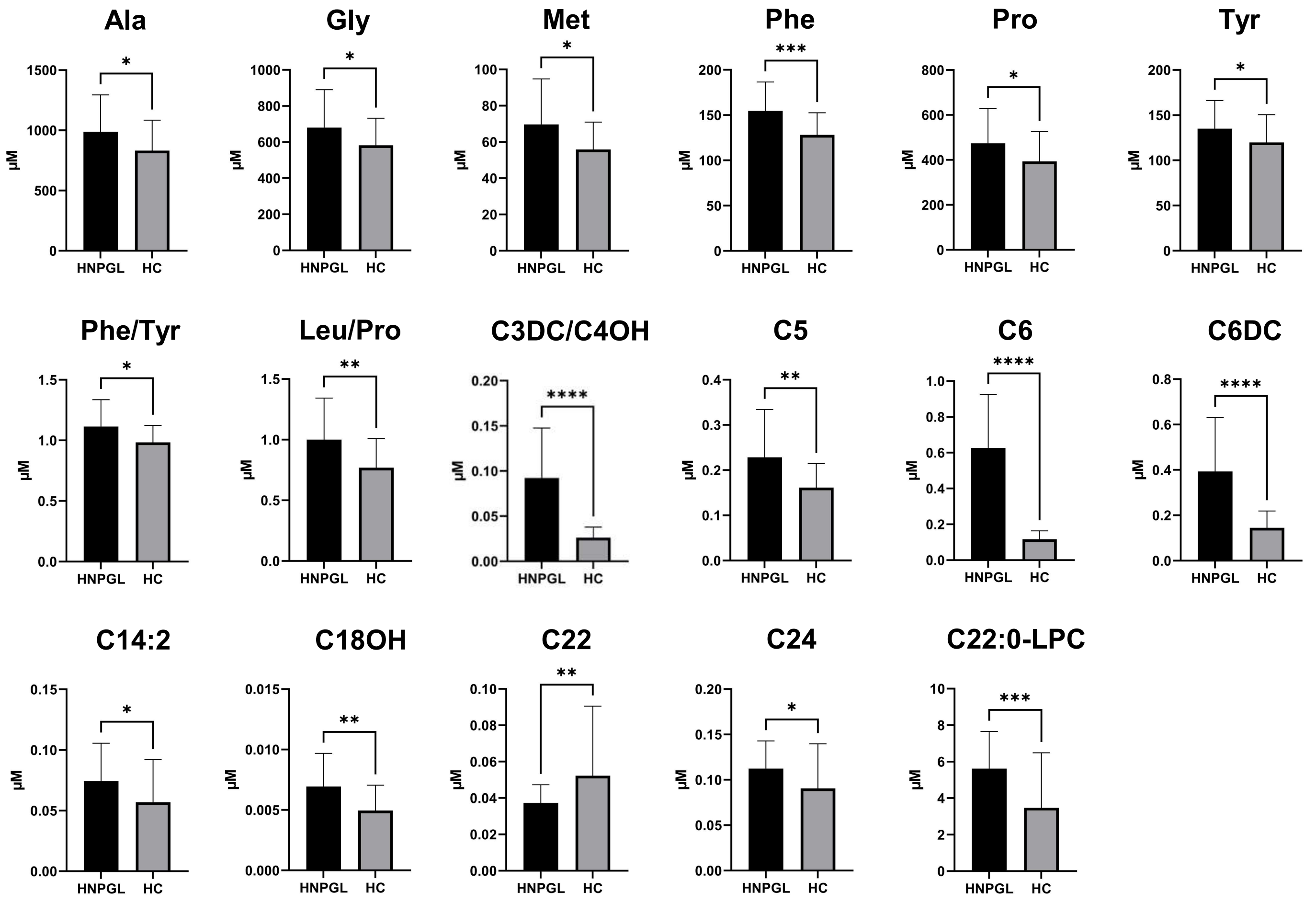

Supplementary Figure S3. Bar charts for the metabolites with VIP values < 1 that showed significantly different plasma concentrations in the head and neck paraganglioma patients (HNPGLs) versus the healthy controls (HCs), including amino acids (Ala, Gly, Met, Phe, Pro, Tyr, Phe/Tyr, Leu/Pro), acyl-carnitines (C3DC/C4OH, C5, C6, C6DC, C14:2, C24, C18OH, C22), and lysophosphatidylcholines (C22-0-LPC). Significance was established by t-test (*p < 0.05; **p < 0.01; ***p < 0.001; ****p < 0.0001).

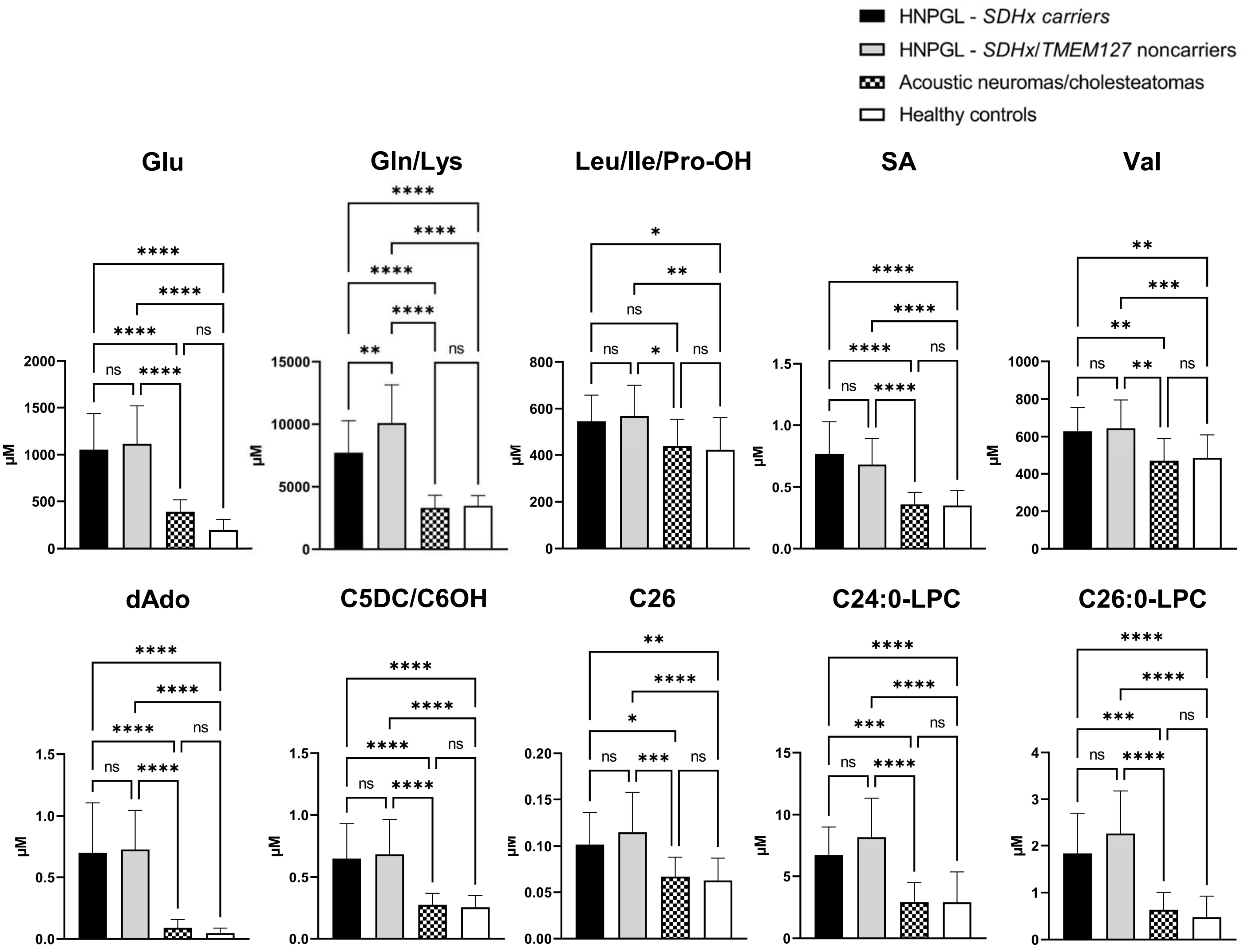

Supplementary Figure S4. Bar charts for Gln/Lys, Glu, Leu/Ile/Pro-OH, SA, Val, dAdo, C5DC/C6OH, C26, C24:0-LPC, C26:0-LPC in the *SDHx* carrier and *SDHx*/*TMEM127* noncarrier HNPGL patients, acoustic neuroma/cholesteatoma (AN/CH) patients and healthy controls (HCs). Significance was established by ANOVA (*p < 0.05; **p < 0.01; ***p < 0.001; ****p < 0.0001).
